# Vital Phenomena

**Published:** 1871-05

**Authors:** J. F. Sanborn


### VITAL PHENOMENA.

BY J. F. SANBORN, M. D.

We read that God made the earth out of chaos, which is the chaos from which all organic substances have their origin, and to which they must all return.

Man stands pre-eminently at the head of creation, and is the being for which all other things were made.

What is the difference between a man while living, and after he is dead?

It is life. What is life?

What is death?

Is it the presence of the soul in the body, that constitues this difference? Is it the presence of a soul in an animal that makes the distinction between a living and a dead animal?

Is the soul and the mind the same, and is that the vital importance? If so, is the idiot destitute of the thinking power, is he a living being? Or is the animal, because it has a mind, therefore, equal with man?

Neither the presence of the soul or the mind, constitutes, physiologically speaking, animal life.

Man in the beginning, was created in the image of his maker.

In what sense was he created in the image of God?

First. God is a trinity. The Father, the Son, and the Holy Spirit; these three are one God.

Man is a trinity, composed of body, mind, and soul; physical, mental, and moral, and it requires all three to make a perfect man.

An animal has the physical and partial development of the mental faculty.

Secondly. Man was created in the image of his Maker, in *contradistinction* to every other thing He had created, and had endowed with life. Man was created PHYSICALLY as well as *morally*, IMMORTAL. Geology teaches that animals died before man was created.

Man in his primeval purity, man as an immortal being, was necessitated to eat and drink, to sustain life, then, as now, but with this difference—those actions that go to make up animal life were in perfect balance.

Undoubtedly, God explained to Adam his physiological, as well as his moral nature; that his animal or physical life consisted of a perfect balance, being maintained in those changes that are continually taking place, and embraced under the four classes of vital changes, known as alimentation, assimilation, disintegration, and depuration. He taught him what food would meet these conditions, and of such he might freely eat, such aliment is represented under the figure of the tree of life.

God taught him that so long as he confined himself to such food, a perfect balance should continue indefinitely in the vital changes, and he should always be in the prime of life, and sickness, pain, or death would have been impossible.

God also taught Adam what class of matter was not food, and was represented under the figure of the tree of knowledge; and He explained how by the use of such matter, the perfect balance in the vital changes that constitute "life" would be destroyed, and by being destroyed, would sometime cease.

The difference, physiologically, between a man living or dead, is in the former the vital changes are taking place, but in the latter they have ceased.

Life, then, is the sum of all the vital changes, and death their suspension. Life is action, death inaction.

In life there is a continual change of matter as food, to the living tissues, and from the living tissues back to the chaos from whence the food was derived.

Death is the suspension of such changes.

God is His own interpreter, and He has made it plain.

Adam by his first transgression, fell physically, as well as morally; disease, pain, and death are the result of this loss of balance in the vital changes.

This condition exists in us by nature, but more so by our practice, for we physically as well as morally follow the teachings of a second lesson, taught Adam by a very different personage from the Divine Creator.

He said to Adam, eat that which God has forbidden, that which He has said is not for food, and which He has said tends toward death; eat that which pleases the eye and gratifies the taste, for ye shall not surely die.

The Liebig theory, that life is a series of chemical changes, wherein matter becomes and ceases to be of the cell structure of animal life, is not in accordance with our ideas of things, but rather there is a vital principle, that elevates and controls matter, with which it becomes associated, in accordance with certain fixed laws, which may be denominated the "LAWS OF LIFE."

Some authors claim that the controlling agency of vital action is electricity, and there are some grounds for such a belief, but of this "let everyone think as he pleaseth."

When vital action ceases, chemical action begins; when chemical action begins, vitality ceases.

Chemical and vital action are incompatible. Vital action may not be in violation of chemical law, but it is higher, and tends upward in its action; while chemical action is downward towards the inorganic condition of matter.

The chemical constituents found in vegetable and animal tissues, are very different in their structures, as found in organized matter, from what they are after they have passed through chemical decomposition.

Take for illustration, sacharine matter, as found in the fruits or cereals. It not only pleases the taste, but it nourishes—it is food; the chemical decomposition of sugar breaks down the cell structure, and separates it into carbonic acid gas and alcohol.

Sugar will digest and nourish, but alcohol will not; it is alcohol when received, it is alcohol in the system, and it is alcohol when it leaves it. It is like the prince of evil. There are no circumstances when you can turn him to any good account. He is evil, and not only evil, but that continually, and so of alcohol. It is broken down matter, and there are no circumstances in which it bears a natural relationship to vitality.

It will do to dissolve gum for varnish, or to burn in our lamps, or to preserve dead bodies in, but never should come in contact with living tissue, for it is antagonistic to vital action.

The continued chemical decomposition of alcohol, does not restore it to a normal relationship with vitality.

Vinegar is but *decayed rotten alcohol*, and it bears such an abnormal relationship, that the vital organs refuse to receive it only in the smallest quantity. Men have been known to boast that they could drink a gallon of N. E. rum in a day, without becoming intoxicated.

N. E. rum is 25 per cent. alcohol, this would make a quart of pure alcohol. This was done in the hottest portion of the year, when the pores of the skin were in the most active condition. The alcohol would pass directly from the stomach to the blood, and from there the lungs would remove a large portion of it, the kidneys would remove none of it, and the skin opened all its portals to allow it to pass. The brain and nerves would feel its hardening influence, which is as true of the living brain as that of the dead; and the finer feelings of those who use it become blunted as the brain becomes hardened. Every muscular fibre with which it comes in contact, feels its influence as a sensitive horse feels the whip, and the vital phenomena is abnormal.

And as the prince of darkness is given to false representation, not to use a more severe term, so alcohol is one of the agencies he uses to delude men to his snare.

But where is the man that can use a pint of vinegar a day, in any part of the year?

The blood is alkaline in its reaction, vinegar in common with all the inorganic or chemical acids, bears an antagonistic relationship to this condition, and it is very destructive to the blood corpuscles.

The acid that the system demands, must in common with all other articles of food, be of organic structure, that is, an acid of growth, as that found in all acid fruits.

The acid of the lemon when chemically decomposed, is known as citric acid, but the acid of the lemon, and the citric acid of commerce, bears the same relationship to each other, so far as vitality is concerned, as living and dead matter.

Weariness, resting and sleep, are among the vital phenomena that demand our attention.

A muscle is composed of a multitude of cells lying side by side, and joined end to end, the latter constitute the fibrilla or filament of a muscle. The fibrilla lying side by side, are separated by a fine gauze like structure, known as connective tissue. The fibrilla with the connective tissue go to make up a muscle.

The cells of the fibrilla are composed of a capsule, and the contents of the cell.

The power of a muscle to contract, is in the contents of the cell; and every action is accompanied with a breaking down of some cells.

Such matter having performed the end of its existence, bears an abnormal relationship to the surrounding living tissue; and if not removed, acts as a poison to it.

In animal life, between living and dead matter, there "is a great gulf fixed," however much may be the passage from the former to the latter, there is no passage from death to life.

Broken down cell structure, is as repugnant to the living tissues, as a corpse is to a living refined community.

Their immediate removal is essential to health, and the best interests of society.

Such matter is removed directly to the blood, and from there the various organs remove it—this action is depuration.

Exercise, within proper limits, and sunlight are the agencies most essential to health on account of their aid to depuration, and in many, if not all diseases, sunlight is one of the most important of all the hygienic remedies. Sunlight is as necessary to animal life, health and growth, as it is to the vigor and health of vegetation.

Let us have plenty of sunlight.

If in muscular action, the breaking down goes on faster than it is removed, it must accumulate as rubbish, and prevent free action.

This condition of things constitutes weariness.

Resting is the suspension of muscular action, which breaks down, until those already broken down are removed. This restoration in the balance, is the condition known as resting.

The continual breaking down and removal of a cell here and another there, without having them renewed, would soon stop all further action for want of whole fibres to contract.

When there is sufficient breaking down for nature to demand repairs, the condition is very different from that which is weariness; weariness is like rubbish in the way. This is like a want of tools to work with—a condition of exhaustion, and in a normal condition of things, is followed by a desire for sleep.

*Natural sleep is the* SUSPENSION of all *action* of *wakeful hours*, and the *concentration of all the vital energies to the* RE-CONSTRUCTION *and repair wherever it is wanted. Nutritious matter in the blood is changed into tissues of the body, and becomes a part of the living structure.* This reconstruction and repair of waste, is assimilation, *and takes place perfectly only during sleep.*

Stupor, that is occasioned by poisoning with alcohol or opium, is no more like true natural sleep, than counterfeit money is like the genuine; it may deceive those who do not know the difference, but at the bank, the headquarters of vitality, none but the genuine can pass.

Digestion of food and good natural sleep, can not both take place in a perfect manner at the same time, and if both actions go on at the same time, both will be imperfectly performed; dreaming and disturbed rest are the consequences, so

"To be easy all night,
Let your supper be light,
Or else you will complain
Of a stomach in pain."

Sleep is the grand restorer of brain, nerve and muscular energy. Take plenty of sleep, and say with Sancho Panza: "Blessed be the man who first invented sleep," but avoid the latter part of the quotation: "But cursed be the man with curses deep, that invented early rising," for one hour of sleep in the first part of the night is worth two after sun up.

The theologians are forever preaching against following nature. But the physiologist will say follow nature, the closer the better, and in no one thing more so, than in taking plenty of sleep in the natural hours for it. Those who wish to sleep in the middle part of the day, should do so before dinner, instead of after it. This is especially true of invalids.

Sleep repairs the waste tissue, in doing so it uses the nutritious matter stored in the blood; if from any cause we loose our usual amount of sleep, the blood is not impoverished by the removal of its nutriment, and there is not the demand for its accustomed supply—if food in the usual quantity is taken, under such circumstances, a rush of blood to an exhausted brain and a headache is the result.

Eating and sleeping should always be in perfect balance.

The wakefulness incident to sickness, is occasioned by the vital action being employed in removing broken down abnormal matter, necessary to restore the lost balance, and there is proverbially a loss of appetite, and the idea conveyed to the Doctor in an answer to his question: How is the appetite? is the system resuming its balance? the appetite being the thermometer, is often by the use of condiments caused to rise much above its normal condition, and a false information is the result. Do not loose sight of the idea that in sickness the action is in depuration, and not in assimilation.

Do not stimulate the appetite nor give food unless it is demanded by the cravings of a normal desire for food.

The first transgression, we read, was in indulging the appetite, which propensity has been handed down from generation to generation, till it may truly be said to be the great sin of the race.

Nutrition should be in exact proportion to the wants of vitality for growth, and to supply the waste, if it is much in excess of these, disease is the result.

The vital phenomena of "taking cold," is worthy of our consideration.

There are five outlets for waste matter, the bowels, the kidneys, the liver, the lungs, and the skin.

In order to perfect health these must be in full action, each doing its full duty up to the full demands of the system.

Taking cold is the inaction of the skin. The waste that should be expelled through it is retained in part, and acts as a poison, aches and pains are the result.

Good reasoning would indicate for the cure, that the skin increase its action until the balance is restored. Water is the great solvent of all organic matter, be it vegetable or animal.

Water is the vehicle by which all food is reduced to a condition that it can be used, and carried to where it is wanted to build up the waste tissues. It is also the vehicle to dissolve, to wash out, and purify the system of all waste matter.

Water, pure and free as God gives it to nourish his creatures and beautify his footstool, bears a natural relationship to vitality; it is kindly received and appropriated by it.

A warm bath is natures own remedy—it opens the pores of the skin, and increases its action, and aids it in its efforts.

A fresh animal membrane tied over the mouth of a phial containing salt water, and thus prepared, inverted in a glass of pure water, just deep enough to cover the membrane, will, in a short time, occasion the water in the glass to become as salt as that in the phial; the quantity of water in the phial will not be changed. Thus water applied to the skin, either in a warm bath, or warm wet cloths, will remove effete matter, and the cloths or bath will be filled with it, in the same manner as the pure water in the glass, is filled with the salt; and that too without occasioning exhaustion of vitality.

One organ acting for another, is known as doing vicarious duty. The inaction of the liver is often followed with sore eyes. The eyes for the time being, doing duty for the liver, to remove from the system that which should be removed by the liver.

The kidneys may, and often do vicarious duty for the skin.

Constipation of the bowels is often accompanied with effete breath, or sore eyes.

The administration of a cathartic would occasion the bowels to remove the offending matter, or a diaretic, the kidneys. But in such a course, aiding nature is not a "*grave*" error. The modus operandi of a cathartic, is readily perceived when viewed from the standpoint that all action is on the part of vitality, and that the cathartic like food is acted on by the vital organism.

The normal action of the small intestines, is to pass nutritious matter from within out, and on to the blood. They are lined with villi, so abundant as to amount to from 90 to the square inch in the upper portion, to 40 in the lower, aggregating 4,000,000.

The digested food does not act on the villi, but the villi act on the food, absorbing it as passing it along.

The cathartic does not act; it too is acted on by the villi, but instead of absorbing as in the case of the food, it is rejected as matter bearing an abnormal relationship, it occasions an antagonistic action.

Antagonistic action, whether of a nation, or of vital organism, is always exhausting in its character; a draft on the capital stock.

The villi not only refuse to absorb the abnormal matter, composing the cathartic, but they reverse their action instead of absorbing and passing it along as is the case of food; it is rejected, and nutritious matter that should pass on and nourish the body, is returned to the bowels, diluting the cathartic, thus protect themselves against its abnormal presence and unnatural influence, and it is hurried out of the system, each part lending a helping foot to get rid of it.

This is choosing between two evils, and curing one disease by producing another—which is false in theory, false in practice, and a "*grave*" fitting error.

### RECAPITULATION.

Life is action, the sum of the vital changes.

Health, their perfect balance.

Cause of disease, the loss of balance.

Disease, natures effort to restore the balance.

Death, the suspension of all the vital changes.

All matter entering the system should be organic, and in its highest degree of development, and should under all circumstances of life, bear a natural relationship to the vital changes, and should add to the motive that propels the wheel of life.

Inorganic matter, and such as has had its organic character changed by chemical action, do not act on the vital machinery any more than does food, but it is acted on and occasions antagonistic action, and acts like a brake on the wheel of life.

All medicines are poisons, occasioning antagonistic action, they diminish an action in one part by increasing it in some other; but not in harmony with vital effort to restore the balance, but "cure one disease by producing another." They are a brake on the wheel of life.

In treating disease, see that all the outlets for waste matter are in full natural action, and restore the vital balance by the use of such remedies as bear a natural relationship to vitality. Remembering that natures law of cure, like natures law of health, is TRUE OBEDIENCE TO PHYSIOLOGICAL LAW.

